# Protist Community Grazing on Prokaryotic Prey in Deep Ocean Water Masses

**DOI:** 10.1371/journal.pone.0124505

**Published:** 2015-04-20

**Authors:** Emma Rocke, Maria G. Pachiadaki, Alec Cobban, Elizabeth B. Kujawinski, Virginia P. Edgcomb

**Affiliations:** 1 Life Science Department, Hong Kong University of Science and Technology, Hong Kong SAR; 2 Geology and Geophysics Department, Woods Hole Oceanographic Institution, Woods Hole, MA, United States of America; 3 Falmouth Academy Internship Program, Falmouth Academy, Falmouth, MA, United States of America; 4 Marine Chemistry and Geochemistry Department, Woods Hole Oceanographic Institution, Woods Hole, MA, United States of America; Laval University, CANADA

## Abstract

Oceanic protist grazing at mesopelagic and bathypelagic depths, and their subsequent effects on trophic links between eukaryotes and prokaryotes, are not well constrained. Recent studies show evidence of higher than expected grazing activity by protists down to mesopelagic depths. This study provides the first exploration of protist grazing in the bathypelagic North Atlantic Deep Water (NADW). Grazing was measured throughout the water column at three stations in the South Atlantic using fluorescently-labeled prey analogues. Grazing in the deep Antarctic Intermediate water (AAIW) and NADW at all three stations removed 3.79% ± 1.72% to 31.14% ± 8.24% of the standing prokaryote stock. These results imply that protist grazing may be a significant source of labile organic carbon at certain meso- and bathypelagic depths.

## Introduction

The deep ocean, more specifically the mesopelagic (200–1000m) and bathypelagic (1000–4000m) depths, are realms of significant remineralization of organic matter, long-term carbon storage and burial [1]. Due to biological processes such as primary and secondary production occurring in epipelagic depths, organic carbon is exported to depth through vertical fluxes of settling particles (particulate organic carbon, or POC), migration of plankton, and physical processes such as the movement of major water masses. Collectively, these processes cause deep ocean waters to be the largest oceanic reservoir of dissolved organic carbon (DOC) [2, 3]. Respiration of this pool of carbon in dark pelagic layers accounts for up to one third of oceanic biological CO_2_ production [4, 5]. These deeper waters however are more difficult to study due to logistical challenges and associated expenses. Nevertheless, examination of food web dynamics in the dark ocean is essential in order to properly understand the role of deep ocean waters in marine biogeochemical cycling.

### Protistan grazing

DOC in mesopelagic and bathypelagic waters is consumed primarily by free-living bacteria (e.g.,[6]). Viral lysis and protistan grazing of free-living bacteria in the deep ocean have been shown to be major top-down mechanisms controlling bacterial concentrations, along with bottom-up controls exerted by substrate availability [7, 8]. Apart from transferring bacterial carbon to higher trophic levels, protistan bacterivory releases bacterially-bound nutrients such as nitrogen and phosphorus, making them available for assimilation back into the food web [9]. Protists egest, on average, 10–30% of ingested matter in the form of undigested cell components [10]. As a result, protists can significantly contribute to DOM and POM in the ocean, and shape the chemical environment for marine bacteria.

Only a few studies to date have focused on deep-water eukaryote grazing rates. The most notable study by Cho et al [11] emphasized grazing rates of heterotrophic nanoflagellates (HNF) on bacteria in the epipelagic and mesopelagic zones of the East China Sea in Korea. Despite lower bacterial abundance and production in the mesopelagic versus the epipelagic zone, HNF clearance rates in both layers were similar, and at times grazing rates in the mesopelagic were higher than those in the epipelagic. This introduced the novel hypothesis that HNF grazing of bacteria played a more important role in carbon assimilation than previously expected in mesopelagic waters. More recently, Pachiadaki et al [12] measured the rate of grazing in the East Mediterranean Sea. Similarities with the Cho study included invariant HNF grazing rates in deep mesopelagic and bathypelagic water layers. Increased grazing rates in the deep oxic-anoxic interface compared to deep oxygenated waters were described in Pachiadaki et al [12]. It should be noted that both of these studies used fluorescently-labeled prokaryote tracing techniques and short-term incubations (incubation times of ~1 hour), which according to Vaque et al. [13] tend to result in lower grazing rates in comparison to long-term techniques (incubations times from 12–24 hours).

Heterotrophic nanoflagellates (HNF; ranging from 2–5μm) in particular, have been found to be responsible for most protist bacterivory in the marine pelagic zone, typically increasing with the trophic state of a system [14, 15]. HNF have adapted specific mechanisms for grazing bacterial cells from more dilute environments such as the deep ocean [16, 17]. Three different mechanisms, including raptorial feeding, filter feeding and diffusion feeding have been suggested by Fenchel [18]. All HNF possess flagella, which aside from providing motility, can undulate in many taxa, generating a water current that helps to direct prey into their feeding apparatus. These adaptations allow HNF to move and concentrate suspended food particles, resulting in clearance rates up to 10^5^–10^6^ times their body volume per hour [18]. Grazing rates will be impacted by protist and prokaryote community compositions, as raptorial feeding mechanisms are optimized for specific prey types.

In addition to HNF, larger protist species can graze both smaller protists as well as bacteria. Heterotrophic microplankton, primarily ciliates and dinoflagellates, are well-known major herbivores of marine phytoplankton [19–21]. Some species contribute significantly to bacterivory, primarily through grazing of large bacteria [22]. Other ciliates are able to collect particles smaller than 1 micron with a specialized filter apparatus [23]. Given their significant clearance rates, protists (including HNF, ciliates, and dinoflagellates) likely affect bacterial abundances in deep ocean habitats. The objective of this study was to quantify and compare protist community grazing rates in epi-, meso-, and bathypelagic waters along a transect in the Atlantic Ocean from Montevideo, Uruguay to Bridgetown, Barbados.

## Methodology

### Site descriptions

**Fig 1:**
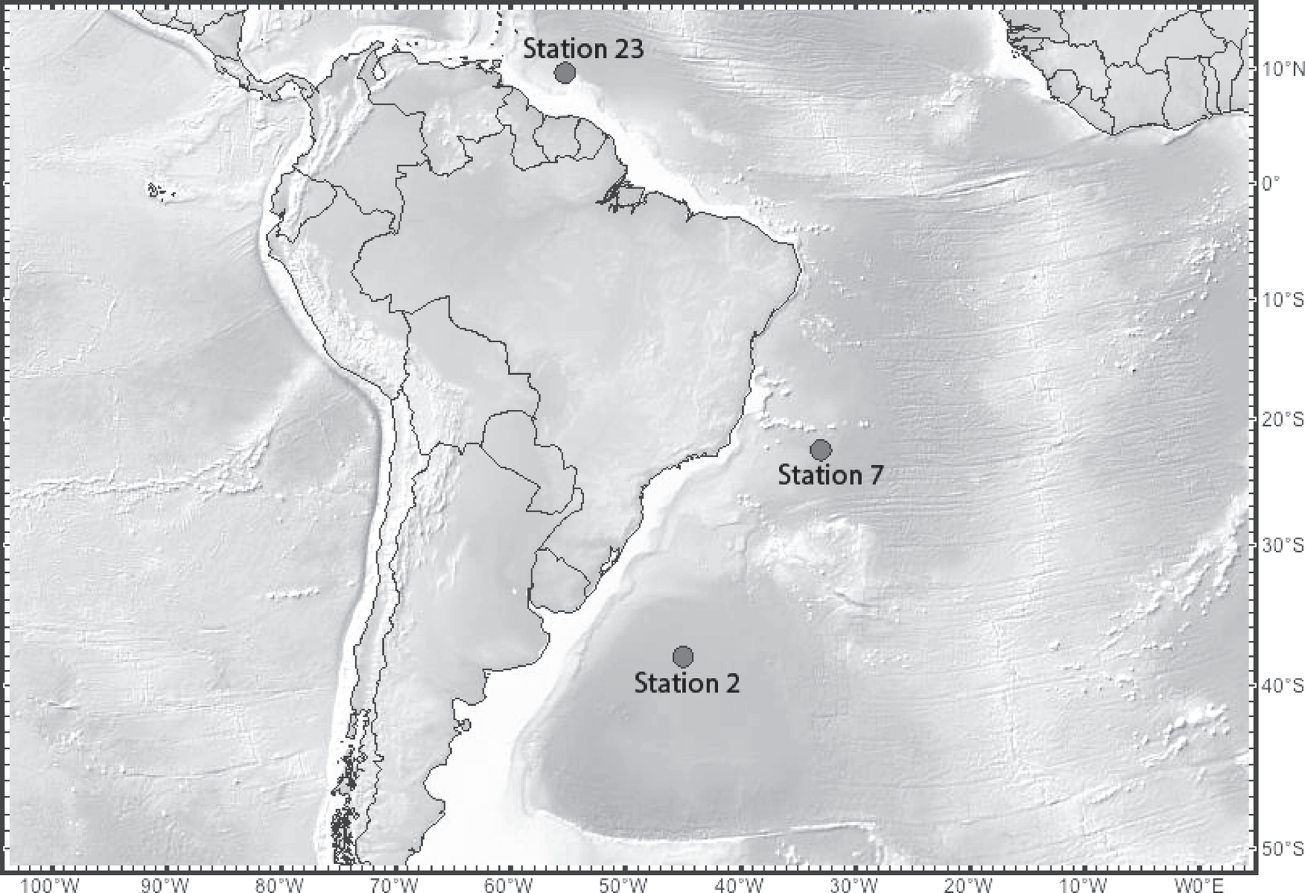
Map of the station locations used in this study, marked as stations 2, 7 and 23.

Incubation experiments were performed during the ‘DeepDOM’ cruise on the R/V *Knorr* from Montevideo, Uruguay to Bridgetown, Barbados between March 25^th^ and May 9^th^, 2013. Grazing experiments were conducted at stations 2, 7 and 23 (station 2: 37° 59’50S, 45°0’W, station 7: 22°29’37S, 33°0’W, station 23: 9° 42’ 1N, 55° 17’57W; Fig 1). Water samples were collected using a SBE9+ CTD rosette with a depth limit of 6000m. A dual SBE3T/SBE4C sensor system augmented by a SBE43 oxygen sensor was used to measure temperature, conductivity, and oxygen. The cruise track covered the equatorial and gyre surface regimes and sampled the deep-water masses of North Atlantic Deep Water (NADW) and Antarctic Intermediate Water (AAIW). Grazing experiments were performed on waters collected from the deep chlorophyll maximum (station 2: 75.8m, station 7: 125.13m, station 23: 65.75m), the upper mesopelagic (250m), AAIW (Stations 2 and 7: 750m, station 23: 875m) and NADW (2500m) at each station (Table 1). Water masses were distinguished from each other through a combination of temperature, salinity and oxygen content. No specific permissions were required for sample collection at these sites, and did not involve any endangered species.

**Table 1:** Water features and metadata (temperature, salinity, dissolved oxygen (DO), total nitrogen (TN), silicate, PO_4_ and NPOC (non-purgeable organic carbon), for Stations 2, 7 and 23.

**Station 2**				
	**DCM**	**220m**	**750m**	**2500m**
**Feature**	Chlorophyll max	Upper mesopelagic	AAIW	NADW
**Temp (°C)**	21.1	15.8	5.1	2.9
**Salinity (PSU)**	36.4	35.7	34.3	34.9
**DO (mL· L** ^**-1**^ **)**	5.2	5.1	5.6	5.4
**Fluor (mg·m** ^**3**^ **)**	0.23	0.0096	0.035	0.059
**TN (μM)**	3.6	8.9	27.2	27.4
**Silicate (μmol·L** ^**-1**^ **)**	0.9	2.2	14.5	50
**PO**_**4**_ **(μmol·L** ^**-1**^ **)**	0.2	0.6	1.9	1.8
**NPOC (μM)**	64.5	58.5	43.5	44.6
**Station 7**				
**Feature**	Chlorophyll max	Upper mesopelagic	AAIW	NADW
**Temp (°C)**	21.8	17.1	5.1	3.1
**Salinity (PSU)**	36.7	35.8	34.3	35
**Fluor (mg·m** ^**3**^ **)**	0.175	0.023	0.017	0.05
**DO (mL· L** ^**-1**^ **)**	5.1	4.9	4.8	5.8
**TN (μM)**	3.9	7.5	33.7	20.4
**Silicate (μmol·L** ^**-1**^ **)**	2.3	3.3	20	28.6
**PO**_**4**_ **(μmol·L** ^**-1**^ **)**	0.2	0.4	1.7	1.3
**NPOC (μM)**	62.2	53	45.2	46
**Station 23**				
**Feature**	Chlorophyll max	Upper mesopelagic	L. mesopelagic/AAIW	bathypelagic
**Temp (°C)**	27.6	15.1	6.5	3.0
**Salinity (PSU)**	36.3	35.7	34.6	35.0
**Fluor (mg·m** ^**3**^ **)**	0.24	0.01	0.031	0.038
**DO (mL· L** ^**-1**^ **)**	4.6	3.2	2.9	6.0
**TN (μM)**	6.4	22	38	21
**Silicate (μmol·L** ^**-1**^ **)**	1.1	8	28	23
**PO**_**4**_ **(μmol·L** ^**-1**^ **)**	0.1	1.3	2.4	1.4
**NPOC (μM)**	73.1	53	48	44

### Preparation of fluorescently labeled prokaryotes (FLP)

To minimize artifacts introduced into grazing studies by using either fluorescently labeled beads or a single cultured organism as a labeled prey species, we prepared fluorescently labeled prey from a mixed whole seawater sample, collected from the Vineyard Sound in Woods Hole, MA, USA. After pre-filtering through 0.8μm pore size filters to exclude protists and metazoa, the sample was used as inoculum into sterile seawater to which 0.1% Marine Broth medium (DIFCO) was added and the enrichment cultures were maintained at room temperature. When they attained exponential growth (as determined by microscopy counts), cells were pelleted by centrifugation (20min at 2000x*g*), re-suspended into sterile seawater and grown for 5 days. The cells were stained with 5-(4,6-dichlorotriazinyl) aminofluorescein (DTAF) as described in Sherr et al. [24] with minor modifications. The prokaryotic enrichments were centrifuged at 14000x*g* for 12min and the pelletized cells were re-suspended in Na_2_CO_3_/NaHCO_3_ buffer (pH: 9.5). The dye DTAF was added at a final concentration of 0.8mg mL^-1^, and the mixture was incubated at 60°C for 3h (vortex mixed every 15min). Staining was followed by three washing steps with Na_2_CO_3_/NaHCO_3_ buffer (pH: 9.5) to remove excess DTAF. Enumeration of prey analogues was performed microscopically. The microscopic observation of the stained cells revealed good staining intensity and absence of cell clumps. They were stored at -20°C and thawed immediately prior to use.

### FLP-based grazing incubations

Prior to conducting all grazing experiments, we first estimated the natural prokaryotic concentration in each seawater sample by fixing a 5mL subsample of target seawater with formaldehyde, filtering through a 0.2μm filter membrane and staining with 1μg mL^-1^ DAPI (4',6-diamidino-2-phenylindole, dihydrochloride). Prokaryotes were counted using a Zeiss Axio Imager M2 epifluorescence microscope.

Once *in situ* prokaryotic numbers were determined, FLP were added at a final concentration of approximately 15% of prokaryotic abundance to 4000mL seawater samples collected at each station from each target depth. Grazing studies were conducted in 4L polycarbonate containers (washed between incubations with 10% HCl and Milli-Q water). The container was gently inverted three times after FLP addition, and two 300mL subsamples were immediately removed and fixed with 30mL 37% formaldehyde (time zero). This was repeated after 24 hours and 48 hours. Containers were incubated at *in situ* temperature and light conditions. All experiments with water from >150m were conducted in the dark in temperature-controlled refrigerators (Table 2). DCM incubations were conducted in on-deck incubators with light shading to reduce ambient light to 10% PAR. Since the concentration of protist predators in different samples could vary, we filtered various volumes (ranging from 5ml to 200ml) of fixed subsamples from each time point for each incubation experiment. Subsamples were filtered onto 0.2μm polycarbonate filters and stored at -20°C until they were counted. Due to time and space constraints on the cruise, we did not perform control experiments to test for loss of labeled prokaryotic prey during shipboard incubations, however we tested this in the laboratory prior to the expedition. Incubations of FLP were conducted in the same types of containers used for shipboard experiments where we filtered out all cells prior to adding FLP (through 0.2μm filtration) and where we filtered out protists (through 0.8 μm filtration). There was no significant change in FLP counts over a 24 hour period.

**Table 2:** Temperatures, light levels and sampling times for grazing experiments at stations 2, 7 and 23.

**Station 2**					
**Depth**	**In situ T (°C)**	**Incubation T (°C)**	**Collection Time**	**FLP added**	**Light**
80 (DCM)	21	15–20	10:45	12:30	20m
220m	15	8	10:45	13:00	Dark
750m	4	4	22:30	00:15	Dark
2500m	<4	4	17:30	20:30	Dark
**Station 7**					
130m (DCM)	21	19–20	14:30	18:00	Dark
250m	16	19–20	8:30	12:00	Dark
750m	5	4	8:30	12:00	Dark
2500m	3	4	8:30	12:00	Dark
**Station 23**					
65m (DCM)	27.6	19–20	21:30	23:00	20m
250m	15.1	11	21:30	23:00	Dark
750m	6.5	4	21:30	23:00	Dark
2500m	3.0	4	7:30	8:00	Dark

FLP = Fluorescently labeled prey (or prokaryotes).

### Epifluorescence Microscopy

Total prokaryote and eukaryote counts for each incubation experiment and time point were conducted using epifluorescence microscopy at 1000x (prokaryotes), and 630x magnification (eukaryotes). All filters were mounted onto slides with immersion oil and observed with a Zeiss Axio Imager M2 epifluorescence microscope. DAPI stained prokaryotes and eukaryotes were enumerated using filter set 49 (ex:465/50, Em: 445/50). FLP’s were counted using Ziess filter set 43 (Ex:550/25, Em:445/50). For each sample, at least 50 fields were counted for prokaryotes and more than 80 fields for protists. A eukaryotic fluorescent in situ hybridization (FISH) probe was applied to confirm the eukaryotic counts (see next section). Two to three replicate counts were performed for each depth and time point. Equations following Salat and Marrasé [25] were used to calculate grazing rates:

G=(FLP_0hr−FLP_24hr)((DAPI_0hr+DAPI_24hr)/(FLP_0hr+FLP_24hr))

where FLP is fluorescently labeled prokaryotes at 0 and 24 hours and DAPI is DAPI stained prokaryotic numbers at 0 and 24 hours.

### Fluorescent In Situ Hybridization (FISH)

Filter pieces were overlaid with 0.2% Metaphore agarose (w/v) in order to avoid cells detaching from the filter during sample processing. Following agarose application, filters were dried at 46°C before further processing. Filter pieces were incubated on slides with pre-warmed hybridization buffer (360μl 5M NaCl, 40μl 1M Tris/HCl, pH = 8, 0.01% detergent, and 25% formamide for specificity) in a 46°C incubation oven in a humidifying chamber. The general eukaryotic probe consisted of three SSU rRNA FISH probes used simultaneously (EUKb mix): E309 (5’- TCAGGCBCCYTCTCCG -3’), E503 (5’- GGCACCAGACTKGYCCTC -3’) and E1193 (5’- GGGCATMACDGACCTGTT -3’) [26].

Following hybridization, filter pieces were retrieved from the slides and washed twice with pre-warmed wash buffer (1mL 1M Tris/HCl pH 8.0, 500μl 0.5M EDTA pH 8.0, and 1490μl 5M NaCl, with ddH_2_O added to 50ml plus 0.01% detergent) for 20 minutes. Filters were then washed in 50ml ddH_2_O for 1 minute, and then briefly washed in 50ml absolute ethanol (96%) before drying. Filter pieces were mounted on slides with 20μl of Citifluor/Vectashield (5.5:1) with 1μg/ml of DAPI. Counts and images were obtained using a Zeiss Axioplan 2 microscope equipped with a Zeiss Axiocam camera.

## Results

**Fig 2:**
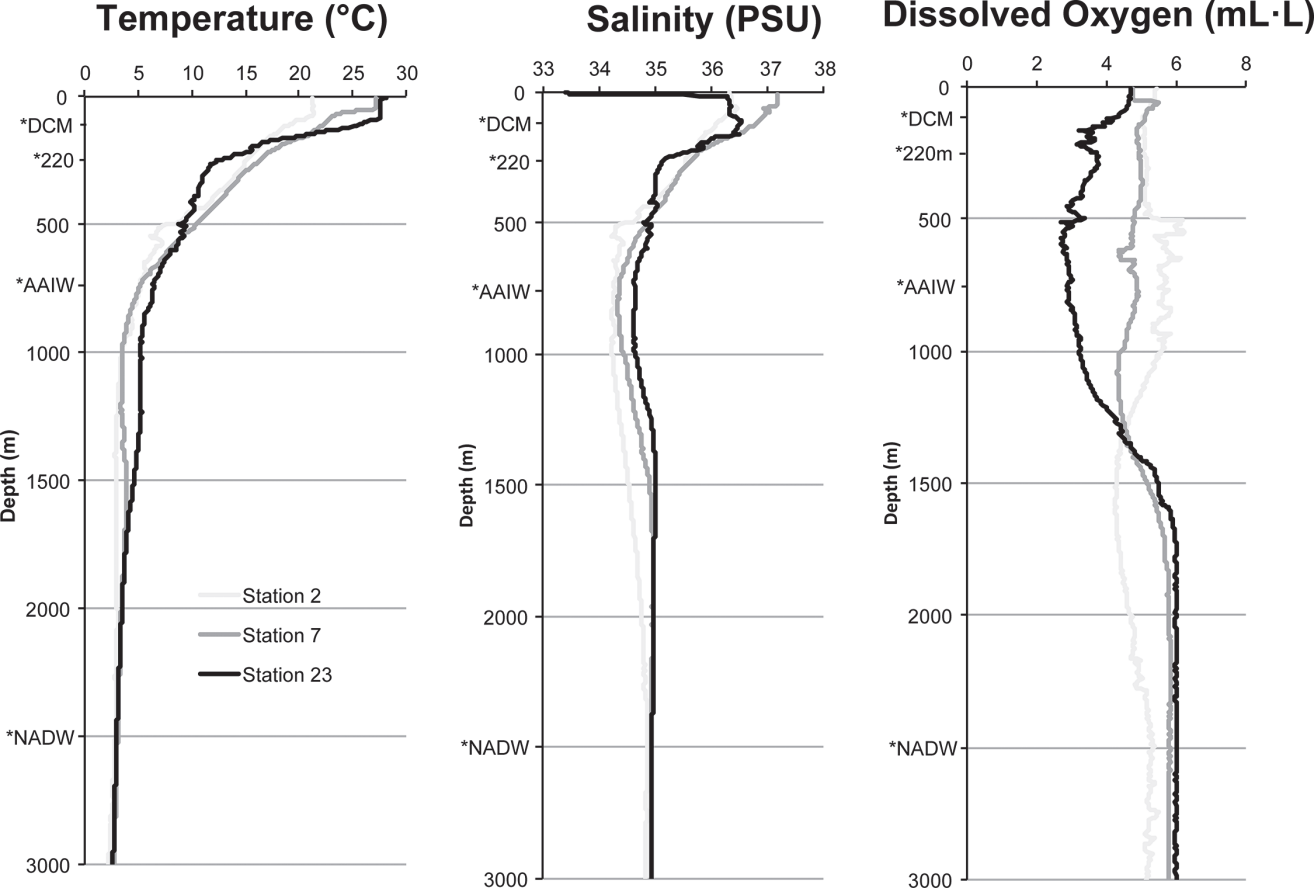
Temperature (°C), salinity (PSU) and dissolved oxygen (mL· L^-1^) depth profiles for stations 2, 7 and 23. Sampling depths are marked with an asterisk.

Temperature, salinity and oxygen profiles for stations 2, 7 and 23 are illustrated in Fig 2. Temperature decreased from 21°C (Station 2) and 27°C (Stations 7 and 23) at the surface down to 100m to approximately 6–7°C around ~600m, after which the temperature declined gradually to approximately 3°C at 3000m. Salinity at all three stations was similar, except for station 23, which had a lower salinity at the very surface (33.4 vs 37, PSU) due to its proximity to the mouth of the Amazon River. Dissolved oxygen profiles varied for each station, but never dropped to hypoxic levels at any of the depths sampled. Nutrient concentrations remained similar through the water column at all three stations, except for total nitrogen (TN) which was higher at the surface waters of station 23, most likely due to Amazon River water input (Table 1).

**Fig 3:**
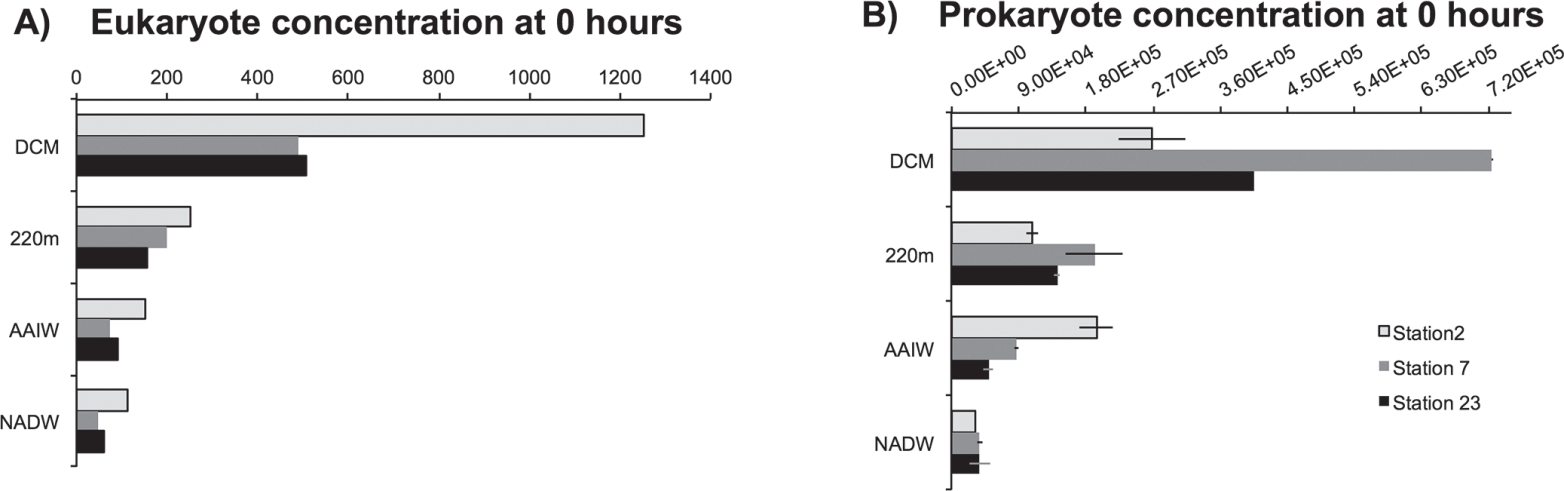
Fig 3A: Eukaryote concentrations (#Euks ml^-1^) at 0 hours obtained through DAPI stained counts at stations 2, 7 and 23. Fig 3B: Prokaryote concentrations (# bacteria·mL^-1^) obtained through DAPI stained counts at stations 2, 7 and 23. Error bars represent the standard deviation of the mean (n = 2).

The *in situ* prokaryote counts (Fig 3 and S1 Table) at time 0 ranged from 2.4 x10^5^ (station 2) to 7.3 x10^5^ cells per mL (station 7) at the deep chlorophyll maximum (DCM), 1.1 x10^5^ (station 2) to 1.9x10^5^ cells per mL (station 7) at 220m, 4.9 x10^4^ (station 23) to 19.4 x10^4^ cells per mL (station 2) at the AAIW, and 3.1 x10^4^ (station 2) to 3.6 x10^4^ cells per mL (station 23) at the NADW.

Eukaryote counts varied for each station and depth (Fig 3 and S1 Table). Time zero counts ranged from 490 (station 7) to 1253 (station 2) cells ml^-1^ in the DCM. At 220m, eukaryote counts ranged from 155–249 cells ml^-1^ (stations 23 and 2). In the AAIW, stations 2 and 7 had 152 and 74 cells ml^-l^, while station 23 had 91 cells ml^-1^. In the NADW, eukaryote cell counts ranged from 49–60 cells ml^-1^ (stations 7 and 23) to 113 cells ml^-1^ (station 2). We initially designed the experiments to run for 24h. Although we suspected that altered water chemistry after 24h might lead to a decline in the eukaryotic population, we decided to add one more time point at 48h in case we were not able to estimate phagotrophy at 24h (if grazing rates were very low). It should be noted that data on the final eukaryotic population size is not necessary for calculating the grazing rates. Because we noted a decline in eukaryote numbers after 24h, we based our phagotrophy calculations on the 24h data. The decline in protist numbers after 48 hours was the most pronounced in samples from the DCM at all stations, dropping by 515, 65 and 238.3 cells ml^-1^ for stations 2, 7 and 23 respectively (S1 Fig). This suggests the appearance of bottle effects that can potentially include grazing of smaller eukaryotes by larger ones, attachment of eukaryotes to container surfaces, and decline in numbers due to altered water chemistry.

Originally, counts of eukaryotes for all samples were performed in triplicate so that standard deviations could be calculated. Following completion of cell counts, it was discovered that a proteinase K step originally incorporated into our FISH protocols caused a significant loss of eukaryotic cells, apparently due to lysis. While this did not impact our counts of prokaryotes, nor our calculations of grazing rates, this would lead to an overestimation of daily grazing activity on a per (eukaryote) cell basis. For this reason we recounted eukaryotes on remaining filter sections for all samples without using proteinase K in the FISH protocol. Unfortunately, remaining replicate filters did not exist for all samples, and for this reason we report eukaryotic cell counts in Fig 3 and S1 Fig from single filters per depth/site.

Eukaryote counts of the second round of FISH filters yielded very similar counts (+/- 8 cells) to DAPI filters. Due to project constraints, specific counts of ciliates were not made, however a higher proportion of ciliate-like cells were observed at DCM and 220m water layers, versus more HNF-like cells in the AAIW and NADW.

**Fig 4:**
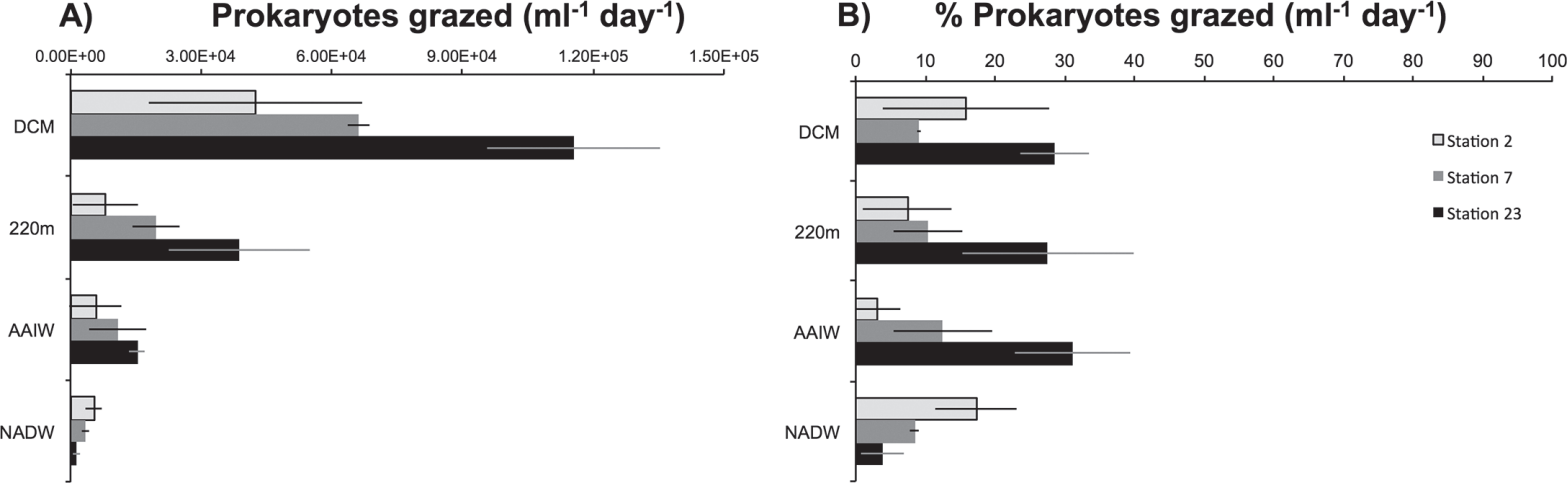
Fig 4A: Community grazing rates of prokaryotes represented as the number of prokaryotes grazed per day. Fig 4B: Community grazing rates of prokaryotes as a percentage of prokaryote standing stock grazed. Error bars represent the standard deviation of the mean (n = 2).

Grazing rates generally decreased with depth at all stations (Fig 4). At specific depths, there were variations in results between different stations. At the DCM, grazing was highest at station 23 at 1.15 x10^5^ cells grazed per day and lowest at station 2 at 4.2 x 10^4^ cells grazed per day. At 220 m, grazing was again highest at station 23 at 3.9x10^4^ cells grazer per day, and lowest at Station 2 at 8.1x10^3^ cells per day. In the AAIW the highest grazing rates were observed at Station 23 (1.5x10^4^ cells per day) and the lowest at Station 2 (5.7 x10^3^ cells per day). In the NADW the highest rates were observed at Station 2 and 7 (5.4x10^3^ and 3.4 x10^3^ cells grazed per day), and the lowest at Station 23 (1.3 x10^3^ cells grazed per day).

### % standing stock of prokaryotes grazed

The percent of standing stock prokaryotes grazed at each station varied with depth (Fig 4). At station 2, 15% of prokaryotes were grazed at the DCM, 7% at 220m, 2% at the AAIW, and 17% was grazed at the NADW. At station 7, 9% was grazed at the DCM, followed by 10%, 12% and 8% at 220m, AAIW and NADW respectively. At station 23, 28%, 27%, 31% and 4% of prokaryotes were grazed at the DCM, 220m, AAIW and NADW respectively.

## Discussion

Only a few studies have focused on grazing in deep ocean waters [11, 12, 27]. Cho et al (2000) and Tanaka et al. (2004) measured grazing at depths down to 500m in the East China Sea and Northwestern Mediterranean, respectively. Recently, Pachiadaki et al. (2014) measured grazing in the deep Mediterranean (3000–3400m depth), however the mesopelagic and bathypelagic waters in this environment have unique features, including anoxic and hypersaline waters. As such, it is difficult to generalize results for all deep bathypelagic waters. Some previous studies have promoted a consistent decrease in grazing impact with depth [28, 29] whilst others show increasing evidence of significant grazing pressure in deep mesopelagic and bathypelagic waters [11, 30, 31]. The present study expands upon previous work by providing further insights into variations in grazing impact in oxygenated deep-water features such as the NADW and AAIW at different stations in the South Atlantic.

Prokaryotic abundance tends to decrease with depth [10, 30, 32, 33]. We found that numbers decreased to 3.0 x10^4^ cells ml^-1^ in the bathypelagic zone. This observation leads to questions about the feeding behavior of protists in these realms. Are they still primarily bacterivores? To what extent do they rely on particulate organic matter? How do they locate their prey in such dilute environments? There are many methods that have been introduced to measure protistan bacterial grazing. A commonly used technique quantifies the disappearance of fluorescently labeled prey analogues. Unrein et al. [34] found that grazing rates were exceptionally similar regardless of the time-scale of the incubation (short- (40 minutes) or long-term (48 hours)). Given the oligotrophic nature of the waters we studied, and the lack of a priori information on composition and abundances of prokaryotic and eukaryotic communities in them, we elected to apply a long-term (24 h) FLP tracer approach here. Despite advantages of this technique in oligotrophic systems, we acknowledge that there are biases inherent in all grazing studies associated with prey species choice when tracking the ingestion of a non-living, fluorescently labeled, mixed prokaryotic community sample. There is conflicting evidence regarding the relative influences of prey surface properties, such as electrical charge [35], surface hydrophobicity [36], chemical cues, as well as motility, size and phylogeny on eukaryotic grazing rates [37–40]. Studies to date seem to emphasize prey size as the factor that most affects grazing, above all other prey properties [37]. Since we didn’t have the opportunity to prepare prey analogues from the targeted water features, we stained cells from a coastal marine site, and thus the results must be interpreted cautiously given that community composition and average cell sizes would not be identical. As FLP prey size was within the size range of observed unlabeled prey communities in our samples on FISH filters (based on DAPI), the effects of FLP size were likely minimal. Additionally, bottle effects must be considered in every incubation study, as these have the potential to affect community composition over the course of the experiment. These effects may be greatest for the deepest samples that experience the largest pressure changes during sample retrieval. Adhesion of the FLP to the walls of incubation bottles was tested prior to the expedition and was not observed to occur. However, we cannot exclude the possibility that FLP may have adsorbed onto large organic particles that may have precipitated.

The deep chlorophyll maximum (DCM) exhibited the highest protistan community abundance of all depths explored. This may be due to the abundance of available nutrients and light at this depth (Table 1). The depth of the DCM varied by station, as it is affected by seasonal upwelling and nutrient fluxes [41]. Eukaryotes in the DCM may be capable of heterotrophy, as well as mixotrophy or autotrophy. Protists are known to consume a high percentage of phytoplankton in marine euphotic zones (from 8 to 131% of phytoplankton stock grazed day^-1^) [21]. The observed decline in eukaryote counts between 0 and 48 hours may reflect death of some fraction of the protist community due to bottle effects or consumption of smaller protists by larger ones. As a result, only the 24-hour filters were used to calculate grazing rates for this study.

Grazing activity (number of prokaryotes grazed per day) in the DCM was the highest out of the four depths examined (Fig 4). A relatively high prokaryote to eukaryote ratio in the DCM may have contributed to high eukaryotic activity there (S2 Fig). When grazing rates were estimated on a per eukaryote basis (prey consumed per eukaryote per hour), rates were not greatly different between the DCM, 220m, and the AAIW (S3 Fig). Bacterivory in the DCM may play a critical role in setting the stage for available substrates for prokaryotes in underlying water masses. Station 23, in particular, exhibited the highest grazing rate at the DCM, a result that might be partially attributed to higher temperatures there [13]. In addition, grazing rates may be higher in the presence of higher non-purgeable organic carbon (NPOC) and nutrient concentrations, due to proximity to the Amazon River plume. High grazing and resulting outputs of faecal material contribute to DOM, POM ([42–45] and dissolved organic nutrients, mostly in the form of ammonium and phosphate [9, 46]. All of this could contribute to the enhanced prokaryote and eukaryote abundances and activity observed in the DCM and upper mesopelagic at station 23 in this study.

One of the most interesting results from this study is the similar percentage of standing stock of prokaryotes grazed daily at depth in comparison to the DCM (Fig 4). Similar percentages of standing stock were grazed in the mesopelagic AAIW and NADW, despite lower eukaryote and prokaryote concentrations (and therefore encounter rates), with the exception of the bathypelagic water mass at station 23 (Figs 3 and 4). Due to technical challenges associated with working with deep-water samples (low concentrations of organisms), the standard deviation of cell counts was high. As a result, the absolute values for % prokaryotes grazed daily should be interpreted with caution. Uncertainty of cell counts was higher for deeper water mass samples where abundances were lowest.

Eukaryote concentrations in this study are comparable to previous studies that have measured grazing down to the mesopelagic zone (Table 3). In contrast, prokaryote-to-eukaryote ratios were considerably lower in this study compared to other water bodies, e.g., Pernice et al [32] (S2 Fig). This could be due to higher numbers of unicellular fungi in waters with higher ratios [32].

**Table 3:** Prokaryote and eukaryote counts and grazing rates reported in the euphotic zone and mesopelagic from different studies.

Reference	Ocean	Depth	Season	Method	Prokaryotic (cells·ml^-1^)	Eukaryotic (cells·ml^-1^)	Grazing Rate (h^-1^)
This study	South/North Atlantic	Epipelagic	March-May	Long term FLP	1.14x10^5^-7.25x10^5^	155–1252	1.3–10.4
This study	South/North Atlantic	Mesopelagic	March-May	Long term FLP	0.54x10^5^-2.1x10^5^	74–152	1.58–6.99
Cho et al. 2000	East China Sea	Epipelagic	April-September	Short term FLP	1.0 – 12x10^5^	0.4–7.5x10^3^	1.5–5.6
Cho et al. 2000	East China Sea	Mesopelagic	April-September	Short term FLP	1.0–2.0x10^5^	0.1–0.4x10^3^	1.0–2.6
Pachiadaki et al. 2014	Mediterranean	Epipelagic	September	Short term FLP	1.11–5.15x10^5^	0.397–1.7x10^3^	5.78–9.05
Pachiadaki et al. 2014	Mediterranean	Mesopelagic	September	Short term FLP	5.4–5.6 x10^4^	15.3–29.7	0.63–0.75
Detmer et al. 1993	Baltic Sea	Epipelagic	July-August	Dilution	3.0–13 x10^5^	500–3500	0.3–0.72
Detmer et al.1993	Baltic Sea	Mesopelagic	July-August	Dilution	~ 4.0 x10^5^	2400	0.017–0.053

As prokaryote production decreases with ocean depth [11, 12, 28], the observed stability of % prokaryotes grazed per day across all depths studied was unexpected. Possible explanations might be linked to the presence of deep-sea particulate organic matter. Recent studies have revealed that macroscopic deep-sea particles are present in large numbers [47]. These particles come from surface water phytoplankton and prokaryotes, which upon cell death, release gel-forming polysaccharides and macro-gels. The formation of these aggregates is greatest within water features with increased biological production, such as interfaces of distinct water masses, e.g., the AAIW and the NADW [48]. The deep-sea environment provides a favorable physical and chemical setting for increased coagulation rates [49]. Large concentrations of aggregates can provide the ideal substrate for bacterial colonization, and serve as point sources of prey for phagotrophic protists which congregate on the surfaces of those aggregates using chemosensory motile behavior [50]. Studies have illustrated that prokaryotes associated with aggregates produce high quantities of extracellular enzymes, which hydrolyze polymers, resulting in a release of DOM. This further stimulates free-living prokaryote populations in those waters [6, 10]. It should be noted that the presence of aggregates in these deeper water layers tends to promote type II statistical errors and between sample variability due to the heterogeneous environment, accounting for the larger error bars here.

## Conclusions

The community grazing rates calculated from this study confirm previous reports of high grazing activity in deep-water masses, and extend our knowledge of grazing impacts into the bathypelagic realm. Eukaryotes in the AAIW and especially NADW/bathypelagic water masses showed consumption of up to 30% of prokaryote standing stock daily. This further illustrates the important role of heterotrophy in community structuring in deep water layers, and potentially biogeochemical cycling through their control of key prokaryotic communities. This study raises interesting questions for future investigations regarding the abilities of protists to locate and consume prey in these very dilute environments.

## Supporting Information

S1 Fig Eukaryote counts (cells ·ml^-1^) taken at 0 and 48 hours. Fig A: Station 2, Fig B: Station 7, Fig C: Station 23.
(TIF)

S2 Fig Prokaryote to eukaryote ratio at 0 hours for stations 2, 7 and 23. Error bars represent the standard deviation of the mean (n = 2).
(TIF)

S3 Fig Grazing rates (prokaryotes grazed per eukaryote per hour) at stations 2, 7 and 23. Error bars represent standard deviation of the mean (n = 2).
(TIF)

S1 Table Table comparing prokaryote and eukaryote counts (at time = 0 hours), community grazing rates and per eukaryote grazing rates. Error values represent standard error of the mean.
(PDF)
