# Supplementary figures and images for: Protist Community Grazing on Prokaryotic Prey in Deep Ocean Water Masses
Source: PLoS One. 2015 Apr 20;10(4):e0124505. doi: 10.1371/journal.pone.0124505 (PMC4404134; doi:10.1371/journal.pone.0124505)

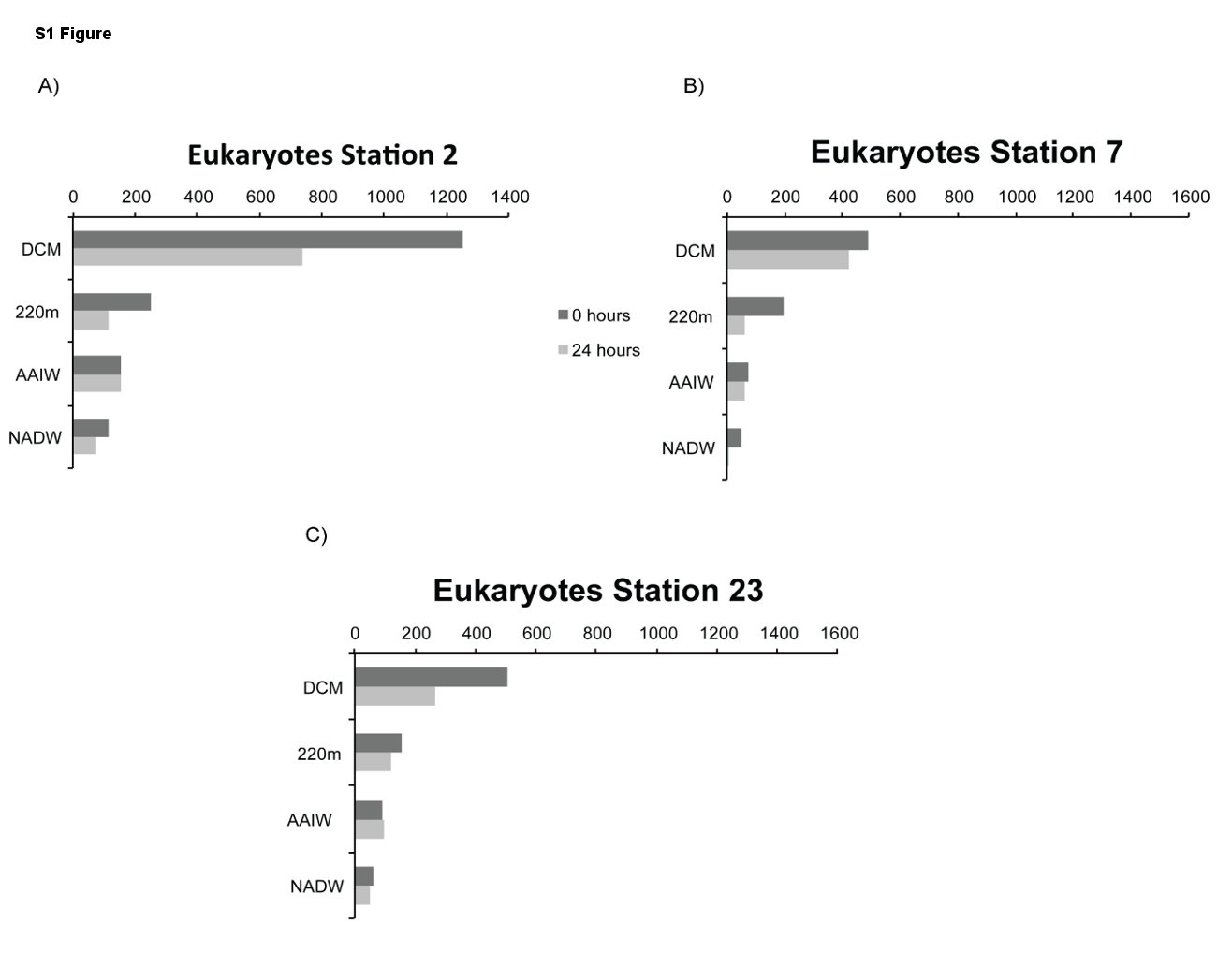

Supplement: S1 Fig — Fig A: Station 2, Fig B: Station 7, Fig C: Station 23. (TIF) [file pone.0124505.s001.tif]

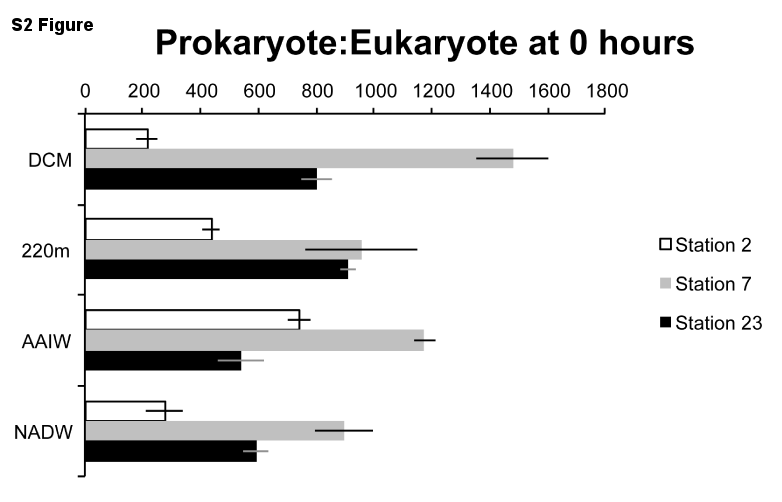

Supplement: S2 Fig — Error bars represent the standard deviation of the mean (n = 2). (TIF) [file pone.0124505.s002.tif]

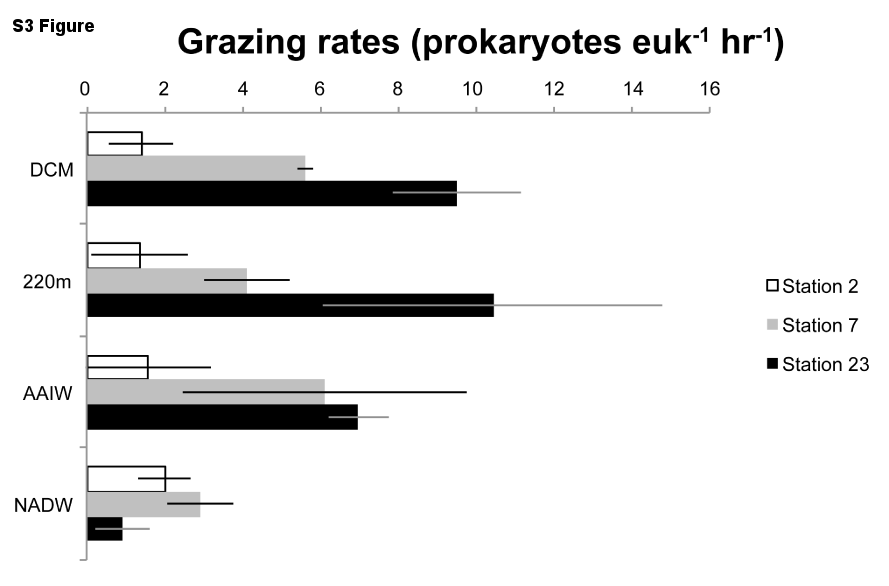

Supplement: S3 Fig — Error bars represent standard deviation of the mean (n = 2). (TIF) [file pone.0124505.s003.tif]
