# Supplementary material for: Protist Community Grazing on Prokaryotic Prey in Deep Ocean Water Masses
Source: PLoS One. 2015 Apr 20;10(4):e0124505. doi: 10.1371/journal.pone.0124505 (PMC4404134; doi:10.1371/journal.pone.0124505)
Supplement: S1 Table — Error values represent standard error of the mean. (PDF) [file pone.0124505.s004.pdf]

S1 Table

Station	Depth	Prokaryotes (cells·ml^{-1})	Eukaryotes (cells·ml^{-1})	Grazing rate (h^{-1})	Grazing rate (prok·euk^{-1} hr^{-1})
2	DCM	$2.68x10^5 \pm 4.56x10^4$	1252	$4.23x10^4 \pm 2.45x10^4$	6.07 ± 3.51
	220m	$1.04x10^5 \pm 7.35x10^3$	249	$8.1x10^3 \pm 7.46x10^3$	5.41 ± 4.98
	AAIW	$1.78x10^5 \pm 2.2x10^4$	153	$5.78x10^3 \pm 5.92x10^3$	4.15 ± 4.25
	NADW	$3.16x10^4 \pm 1.34x10^2$	113	$5.45x10^3 \pm 1.85x10^3$	9.02 ± 3.06
7	DCM	$7.24x10^5 \pm 1.41x10^3$	490	$6.62x10^4 \pm 2.39x10^3$	13.52 ± 0.49
	220m	$1.91x10^5 \pm 3.85x10^4$	199	$1.97x10^4 \pm 5.33x10^3$	14.05 ± 3.8
	AAIW	$8.7x10^4 \pm 2.83x10^3$	74	$1.09x10^4 \pm 6.49x10^3$	20.89 ± 12.45
	NADW	$3.78x10^4 \pm 3.96x10^3$	49	$3.47x10^3 \pm 1.0x10^3$	10.03 ± 2.9
23	DCM	$4.05x10^5 \pm 9.2x10^2$	505	$1.15x10^5 \pm 1.98x10^4$	24.04 ± 4.13
	220m	$1.41x10^5 \pm 4.34x10^3$	155	$3.89x10^4 \pm 1.62x10^4$	27.48 ± 11.45
	AAIW	$4.92x10^4 \pm 7.35x10^3$	91	$1.53x10^4 \pm 1.72x10^3$	24.93 ± 2.8
	NADW	$3.82x10^4 \pm 1.38x10^4$	61	$1.36x10^3 \pm 1.0x10^3$	3.86 ± 2.84
